# Supplementary material for: Healthy ageing of cloned sheep
Source: Nat Commun. 2016 Jul 26;7:12359. doi: 10.1038/ncomms12359 (PMC4963533; doi:10.1038/ncomms12359)
Supplement: Supplementary Information — Supplementary Figures 1-2, Supplementary Tables 1-6 and Supplementary References [file ncomms12359-s1.pdf]

Supplementary Information

Supplementary Figure 1. Schematic for radio-telemetric measurements of blood pressure in sheep.

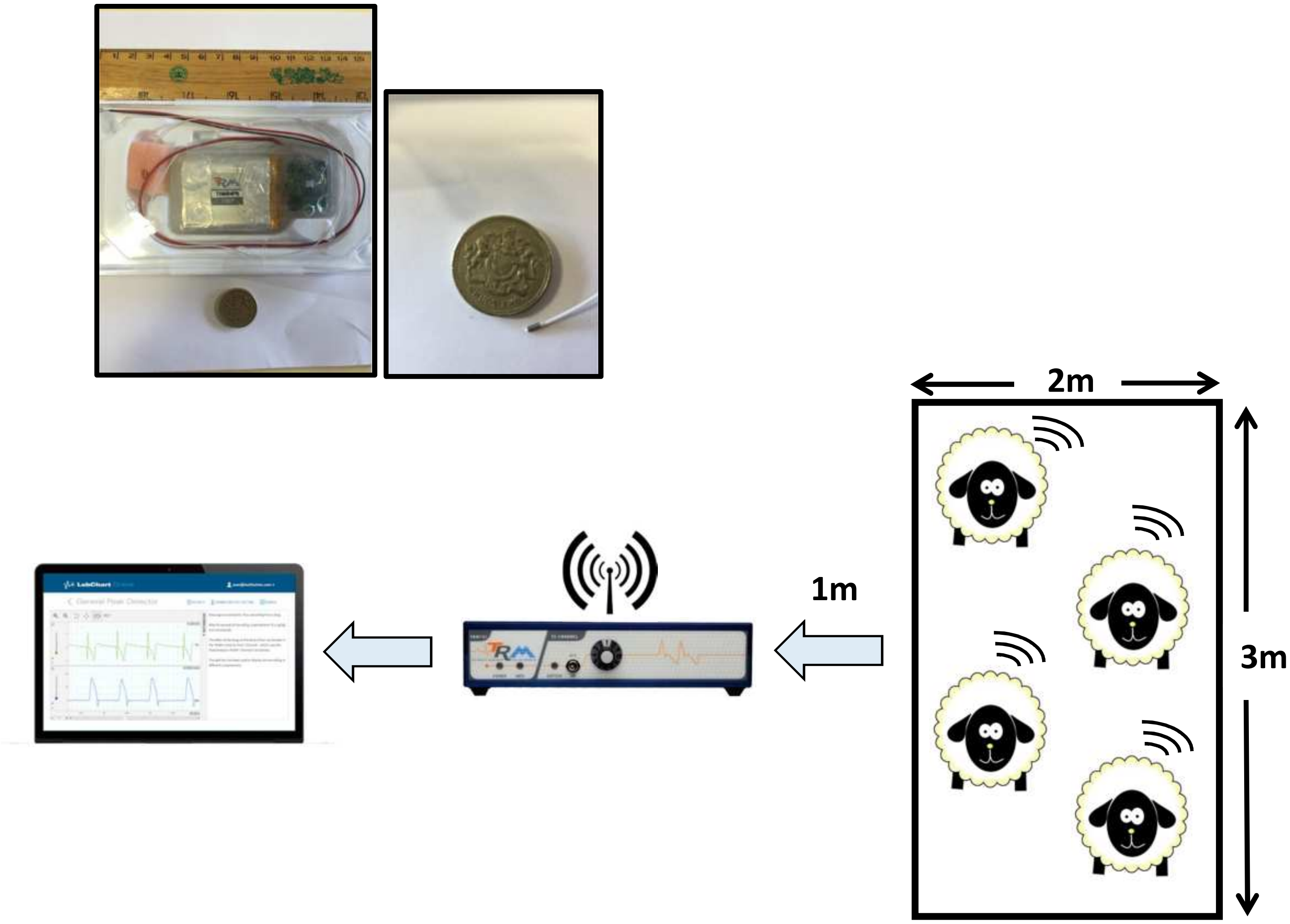

Cardiovascular data were recorded using solid-state pressure radiotelemeters (TRM84PB; Telemetry Research, distributed by Millar Ltd, Oxon, UK) inserted, during general surgery, into the carotid arteries of four sheep at a time. Probe body **(a)** and solid-tip **(b)** shown relative to a UK pound coin. Telemetry Research probes allow for continuous (24h/7d) recording of individual animals, housed as a group **(c)**, in their home environment (e.g. a floor pen approximately 6m^2). This minimises stress-related artifactual elevation of blood pressure. Wireless recording at 2kHz (i.e. beat-to-beat) continued for 3-4 days for each sheep, with data received by an automated scheduler pod approximately 1 m outside the pen **(d)**. The receiver automatically downloaded data to acquisition software on a laptop **(e)** (LabChart 7; AD Instruments, Oxon, UK). Minute means (after exclusion of artefacts) were subsequently derived using MS Excel. After experiments were ended, telemeters were removed from all sheep by general surgery. The cardiovascular protocol was successfully completed for all sheep tested (4 Finn-Dorset and 6 Lleyn clones, and 11 ET-Controls).

Supplementary Figure 2. Images show ventrodorsal radiographic views of the hip joints of three healthy sheep aged 5 years, enrolled in an unrelated study at the Royal Veterinary College and University College London, UK (courtesy of Professors Gordon Blunn and Allen Goodship). In all cases, the femoral heads appear normally seated within the acetabulae, with minimal remodelling. The variable degree of increased bone density around the acetabulum is highlighted with arrows.

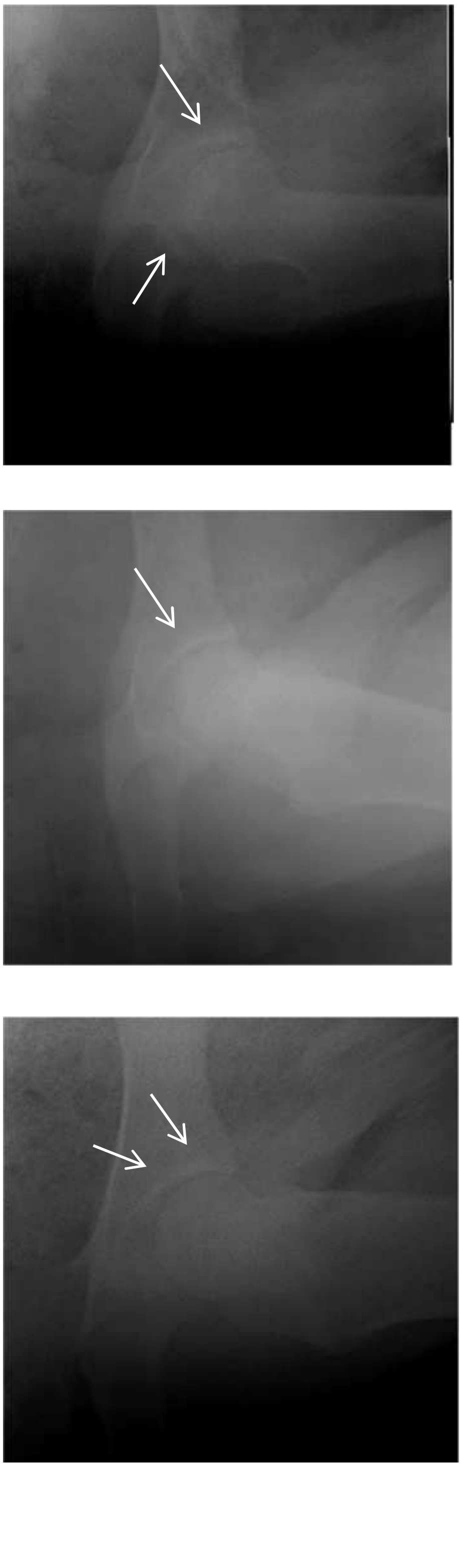

Supplementary Table 1. Microsatellite analyses of cloned sheep (Finn Dorset, Lleyn, A089 and 2264), nuclear donor cells (OP5D3F3, LFF4, SFF1, SFF5) and Dolly (skin fibroblasts).

Microsatellite marker	No. Alleles	Allele size range (bp)	Clones and cell lines			
			Finn Dorset OP5D3F3 Dolly	Lleyn LFF4	A089 SFF1	2264 SFF5
AE119	6	145-179	175 / 177	158 / 158	145 / 151	151 / 179
CSRD247*	5	223-235	223 / 227	227 / 235	229 / 229	227 / 233
ETH152*	3	186-192	186 / 192	190 / 190	186 / 186	186 / 192
FCB11	6	121-141	121 / 123	123 / 141	121 / 135	131 / 133
FCB20	6	91-115	91 / 95	93 / 95	91 / 107	103 / 115
HSC	5	262-282	268 / 272	282 / 282	272 / 274	262 / 274
INRA005*	6	127-147	127 / 131	127 / 147	137 / 141	131 / 139
INRA006*	5	110-134	110 / 110	124 / 134	110 / 132	116 / 134
INRA023*	4	194-216	198 / 216	194 / 198	206 / 216	206 / 206
INRA049	3	137-159	159 / 159	137 / 159	137 / 137	139 / 159
INRA063*	4	169-177	175 / 177	169 / 177	169 / 177	169 / 171
INRA081	7	178-210	196 / 202	186 / 202	178 / 204	192 / 210
INRA172*	4	126-164	160 / 164	154 / 164	126 / 160	154 / 160
MAF214*	3	187-191	191 / 191	191 / 191	187 / 189	189 / 191
MAF65*	3	124-134	127 / 127	127 / 135	125 / 135	127 / 127
McM042*	3	81-95	87 / 87	87 / 87	81 / 87	87 / 95
McM527*	3	164-180	180 / 180	164 / 176	168 / 172	166 / 174
HH47	4	127-141	127 / 129	133 / 141	133 / 133	127 / 127
HUJ616	3	114-122	122 / 122	120 / 120	120 / 120	114 / 122
MAF70	6	129-153	133 / 137	129 / 147	137 / 137	145 / 153
OarFCB20*	6	89-115	89 / 93	91 / 93	89 / 105	101 / 115
Amelogenin*			x / x	x / x	x / y	x / x

Number of alleles and range in fragment size found within our population are provided in columns 2 and 3. All clones were an exact match to their respective parental cell line.

*Markers recommended by the International Society of Animal Genetics

Supplementary Table 2. Body composition and metabolic parameters of the three single clones with reference to male ET Controls.

**Clone (ID No.)**	**2265**	**2264**	**A089**	**ET Controls**
**Cell Line**	LFF4	SFF5	SFF1	-
**Genotype**	Lleyn	Unknown	Unknown	Suffolk-SBF†
**Sex**	Female	Female	Male‡	Male‡
**No.**	1	1	1	6
**Age, years**	8	7	9	6
**A. Body composition** (DEXA)				(Mean ± SD)
Fat,% body mass	37.8	42.0	23.3	29.1 ± 6.0
**B. Glucose tolerance test (GTT)**				
**i. Glucose**				
Basal glucose, mmol/L ††	4.26	4.88	5.86	3.76 ± 0.24
Peak glucose, mmol/L	20.5	22.0	20.6	19.6 ± 1.4
iAUC, units	778	885	666	682 ± 104
k, % per minute	1.27	1.07	1.05	1.61 ± 0.24
T1/2, minutes	54.6	64.7	66.1	43.9 ± 6.1
**ii. Insulin**				
Basal insulin, µIU/mL	75.0	60.3	52.5	14.2 ± 4.1
Peak, µIU/mL	192	128	65	82.2 ± 21.3
∆Max, µIU/mL	117	68	13	68.0 ± 20.2
iAUC, units	6368	8985	933	5183 ± 2343
**C. Insulin tolerance test (ITT)**				
Basal glucose, mmol/L ‡‡	3.78	4.63	4.98	3.45 ± 0.62
K_{4-16} ,% per minute	2.84	3.74	2.04	4.3 ± 1.8
Half time, minutes	24.4	18.5	34.0	18.0 ± 6.1

† - Genotype: Suffolk x Scottish Blackface
‡ - Sex: all males were entire
†† - Non fasted - i.e. 4 hours after the morning meal
‡‡ - Overnight fast i.e. sampled prior to the morning meal

Supplementary Table 3. Body fat, blood glucose and insulin concentrations in sheep undergoing metabolic assessments in previous studies.

Genotype	Age (Years)	Dietary Status	Age (Years)	Basal insulin (µIU/mL)		Glucose tolerance test					Reference
				Lean	Obese	**Dose** (mg/kg Wt)	**Peak glucose** (mmol/L)		**Peak insulin** (µIU/ml)		
							Lean	Obese	Lean	Obese	
Dorset	5	Fed	5	9	30	350	12	13	50	200	1
Dorset X	2 to 4	Fasted†	2 to 4	12	35	-	-	-	-	-	2
Scottish Blackface	2	Fasted†	2	8	9	500	18	23	50	116	3
Dorset x Greyface	1	Fasted†	1	18	40	500	24	26	80	120	4

† = Overnight fast.

References

1. **Body fat** (Dissection – e,g, Omental fat)**:** Lean = 0.3kg, Obese = 2.7kg
2. **Body fat** (Dissection and chemical analyses)**:** Lean = 25%, Obese = 49%
3. **Body fat** (DEXA)**:** Lean = 17%, Obese = 33%
4. **Body fat** (DEXA)**:** Lean = 26%, Obese = 45%

Supplementary Table 4. Cardiovascular parameters (blood pressure, heart rate) in studies of sheep at different ages

Age	≤1 year[6]	1 year[1]	1 year[8]	≤2 years[2]	≤2 years[7*]	2 years[8]	2 years[9]	3 years[4]	5 years[8]	6 years[*]	7 years[5]	≥8 years[2]
**Pressure (mm Hg)**												
Systolic	100 ± 4	105 ± 2	nd	125 ± 3	115 ± 4	nd	125 ± 3	nd	nd	124 ± 8†	nd	132 ± 3
Diastolic	77 ± 4	78 ± 2	nd	78 ± 2	79 ± 2	nd	92 ± 3	nd	nd	93 ± 7†	nd	86 ± 3
Mean arterial pressure	87 ± 4	89 ± 1	78 ± 2	94 ± 2	91 ± 3	79 ± 2	106 ± 3	70 ± 3	80 ± 1	107 ± 7†	73 ± 5*	101 ± 3
**Heart rate (beats/min)**	107 ± 5	89 ± 2	84 ± 3	nd	73 ± 6	79 ± 3	98 ± 5	60 ± 2	79 ± 3	94 ± 16†	79 ± 10	nd

Table: Data are reported as mean ± SE or mean S.D (†, to indicate SD rather than SE) from cardiovascular studies in control sheep in which resting blood pressure was measured either by direct cannulation (reference: 1, 4, 5, 6, 8), by an implanted radiotelemeter (reference: 7 & 3 (current study)) or indirectly using a sphygmomanometer (reference, 2). nd, not determined. *, determined by telemetry

References:

5.	*n* 6 female sheep:	age, 12 months
6.	*n* 10-15 female sheep	
*Reference population from current study	*n* 11 female sheep	age, 69 months
7.	*n* 7 female sheep	age, 40 months
8.	*n* 6 female sheep	age, 84 months
9.	*n* 6 female sheep	age, 8-12 months
10.	*n* 4 female sheep	age, 8-12 months
11.	*n* 5 female sheep	age, 12, 24, 60 months
12.	*n* 11 female sheep	age, 22 months

Supplementary Table 5. Summary of musculoskeletal assessments.

**Sheep (Genotype) (Age)**	**Clinical Examination** Assessment was made using the seven point scale (0 to 6) developed for assessing posture and locomotion by Kaler *et al* 13.	**Radiographic Assessment** Osteoarthritis was graded using a modified Kellgren and Lawrence14 scale, where 0 = no evidence, 1 = questionable, mild osteophytosis, 2 = moderate osteophytosis and minor bone remodelling and 3 = severe osteophytosis with definite bone remodelling.	
	**Reviewer 1**	**Reviewer 1**	**Reviewer 2**
**2265 (Lleyn) (8 years) Female**	**Scored** 0. No significant musculoskeletal abnormalities identified	**Hips*:** Right (1) Left (1)<br>**Stifles:** Right (1) Left (1)<br>**Hocks:** not taken<br>**Elbows:** Right (0) Left (0)<br>**Carpi:** Right (0) Left (1)	**Hips*:** Right (0) Left (0)<br>**Stifles:** Right (0) Left (0)<br>**Hocks:** not taken<br>**Elbows:** Right (0) Left (0)<br>**Carpi:** Right (0) Left (0)
**2264 (Unknown) (7 years) Female**	Scored 2. Uneven posture, but no clear shortening of stride. Visible nodding of head in time with short stride. Mildly lame on the left foreleg. Bilateral varus of the stifles. Thickening of right elbow and slight lateral deviation of the carpus. Soft tissue thickening of the right hock.	**Hips*:** Right (1) Left (1)<br>**Stifles:** Right (2) Left (1) Distinct, large mineralisation associated with the right stifle.<br>**Hocks:** Right (1) Left (0)<br>**Elbows:** Right (3) Left (2)<br>**Carpi:** Right (0) Left (0)	**Hips*:** Right (0) Left (0)<br>**Stifles:** Right (1) Left (1)<br>**Hocks:** Right (0) Left (0)<br>**Elbows:** Right (1) Left (0)<br>**Carpi:** Right (0) Left (0)
**388 (Lleyn) (7 years) Female**	Scored 0. No significant musculoskeletal abnormalities identified	**Hips*:** Right (1) Left (1)<br>**Stifles:** Right (0) Left (0)<br>**Hocks:** Right (0) Left (0)<br>**Elbows:** Right (1) Left (0)<br>**Carpi:** Right (1) Left (1)	**Hips*:** Right (0) Left (0)<br>**Stifles:** Right (0) Left (1)<br>**Hocks:** Right (0) Left (0)<br>**Elbows:** Right (0) Left (0)<br>**Carpi:** Right (1) Left (1)
**385 (Lleyn) (7 years) Female**	Scored 0. No significant musculoskeletal abnormalities identified, slightly thickened left carpus (soft tissue).	**Hips*:** Right (1) Left (1)<br>**Stifles:** Right (0) Left (0)<br>**Hocks:** Right (0) Left (0)<br>**Elbows:** Right (0) Left (0)<br>**Carpi:** Right (1) Left (0)	**Hips*:** Right (0) Left (0)<br>**Stifles:** Right (0) Left (0)<br>**Hocks:** Right (0) Left (0)<br>**Elbows:** Right (0) Left (0)<br>**Carpi:** Right (0) Left (0)
**386 (Lleyn) (7 years) Female**	Scored 1. Uneven posture, but no clear shortening of stride. Mild swelling of the right foreleg around and distal to the elbow.	**Hips*:** Right (1) Left (1)<br>**Stifles**: Right (1) Left (1)<br>**Hocks:** Right (0) Left (not taken)<br>**Elbows:** Right (1) Left (0)<br>**Carpi:** Right (0) Left (1)	**Hips*:** Right (0) Left (0)<br>**Stifles**: Right (0) Left (0)<br>**Hocks:** Right (0) Left (not taken)<br>**Elbows:** Right (0) Left (0)<br>**Carpi:** Right (0) Left (0)

**382 (Lleyn) (7 years) Female**	Scored 0. No significant musculoskeletal abnormalities identified.	**Hips*:** Right (1) Left (1)<br>**Stifles:** Right (0) Left (1)<br>**Hocks:** Right (1) Left (0)<br>**Elbows:** Right (1) Left (0)<br>**Carpi:** Right (0) Left (0)	**Hips*:** Right (0) Left (0)<br>**Stifles:** Right (1) Left (1)<br>**Hocks:** Right (0) Left (0)<br>**Elbows:** Right (0) Left (0)<br>**Carpi:** Right (0) Left (0)
**389 (Lleyn) (7 years) Female**	Scored 0. No significant musculoskeletal abnormalities identified.	**Hips*:** Right (1) Left (1)<br>**Stifles:** Right (1) Left (1)<br>**Hocks:** Right (0) Left (0)<br>**Elbows:** Right (1) Left (1)<br>**Carpi:** Right (0) Left (0)	**Hips*:** Right (0) Left (0)<br>**Stifles:** Right (0) Left (0)<br>**Hocks:** Right (0) Left (0)<br>**Elbows:** Right (0) Left (0)<br>**Carpi:** Right (0) Left (0)
**387 (Lleyn) (7 years) Female**	Scored 0. Slight thickening of left hock.	**Hips*:** Right (1) Left (1)<br>**Stifles:** Right (1) Left (1)<br>**Hocks:** Right (0) Left (0)<br>**Elbows:** Right (0) Left (0)<br>**Carpi:** Right (0) Left (0)	**Hips*:** Right (1) Left (1)<br>**Stifles:** Right (1) Left (1)<br>**Hocks:** Right (0) Left (0)<br>**Elbows:** Right (0) Left (0)<br>**Carpi:** Right (0) Left (0)
**A089 (Unknown) (9 years) Male**	Scored 0. No significant musculoskeletal abnormalities identified. Thickening of left hock.	**Hips*:** Right (1) Left (1)<br>**Stifles:** Right (1) Left (1)<br>**Hocks:** Right (0) Left (0)<br>**Elbows:** Right (0) Left (0)<br>**Carpi:** Right (0) Left (0)	**Hips*:** Right (0) Left (0)<br>**Stifles:** Right (1) Left (0)<br>**Hocks:** Right (0) Left (0)<br>**Elbows:** Right (0) Left (0)<br>**Carpi:** Right (0) Left (0)
**2260 (FinnDorset) (8 years) Female**	Scored 0. No significant musculoskeletal abnormalities identified. Slight thickening of the R carpus.	**Hips*:** Right (1) Left (1)<br>**Stifles:** Right (1) Left (1)<br>**Hocks:** Right (0) Left (0)<br>**Elbows:** Right (0) Left (0)<br>**Carpi:** Right (0) Left (0)	**Hips*:** Right (0) Left (0)<br>**Stifles:** Right (1) Left (0)<br>**Hocks:** Right (0) Left (0)<br>**Elbows:** Right (0) Left (0)<br>**Carpi:** Right (0) Left (0)
**2263 (FinnDorset) (8 years) Female**	Scored 0. No significant musculoskeletal abnormalities identified.	**Hips*:** Right (1) Left (1)<br>**Stifles:** Right (1) Left (1)<br>**Hocks:** Right (0) Left (0)<br>**Elbows:** Right (0) Left (0)<br>**Carpi:** Right (0) Left (0)	**Hips*:** Right (0) Left (0)<br>**Stifles:** Right (1) Left (1)<br>**Hocks:** Right (0) Left (0)<br>**Elbows:** Right (0) Left (0)<br>**Carpi:** Right (0) Left (0)
**2261 (FinnDorset) (8 years)**	Scored 0. No significant musculoskeletal abnormalities identified.	**Hips*:** Right (1) Left (1)<br>**Stifles:** Right (0) Left (1)<br>**Hocks:** Right (0) Left (0)	**Hips*:** Right (0) Left (0)<br>**Stifles:** Right (0) Left (0)<br>**Hocks:** Right (0) Left (0)

**Female**		**Elbows:** Right (0) Left (0)<br>**Carpi:** Right (0) Left (0)	**Elbows:** Right (0) Left (0)<br>**Carpi:** Right (0) Left (0)
**2262 (FinnDorset) (8 years) Female**	Scored 0. No significant musculoskeletal abnormalities identified other than abnormal cleat on left hind foot	**Hips*:** Right (1) Left (1)<br>**Stifles:** Right (3) Left (1) Small distinct mineralisations associated with the left stifle; larger distinct mineralisations associated with the right stifle.<br>**Hocks:** Right (3) Left (3)<br>**Elbows:** Right (3) Left (2-3)<br>**Carpi:** Right (1-2) Left (3)	**Hips*:** Right (0) Left (1)<br>**Stifles:** Right (2) Left (2)<br>**Hocks:** Right (3) Left (3)<br>**Elbows:** Right (3) Left (3)<br>**Carpi:** Right (2) Left (3)

Hips*: ventrodorsal radiographic views of the hips of all sheep consistently showed a slight increase in bone density around the acetabular rim, which could indicate mild osteophytosis or sclerosis. However no sheep had evidence of osteophytosis or remodelling of the femoral head or neck.

13.

Supplementary Table 6. Pregnancy outcomes following transfer (ET) of SCNT embryos reconstructed from enucleated oocytes that were either untreated or had undergone pre-treatment with caffeine according to the protocols of Choi et al. 15. Donor cell line was OP5D3F3.

Date of ET	No. recipients	Oocyte treatment	Blastocysts transferred	Term pregnancies	Pregnancies with live offspring	Live offspring	Alive at 1 week	Alive at 3 months
30.01.07	5	Caffeine	10	2	1	1	0	0
15.02.07	5	Caffeine	12	3	2	3	2	1
20.02.07	5	Caffeine	13	2	2	3	3	3
01.02.07	4	Control	8	2	0	0	0	0
13.02.07	4	Control	9	1	1	2	2	0
22.02.07	3	Control	6	1	1	1	0	0

Supplementary References

1. McCann J.P., Ullmann M.B., Temple M.R., Reimers T.J. & Bergman E.N. Insulin and glucose responses to glucose injection in fed and fasted obese and lean sheep. *J Nutr.* **116,** 1287-97 (1986).
2. Bergman E.N., Reulein S.S. & Corlett RE. Effects of obesity on insulin sensitivity and responsiveness in sheep. *Am J Physiol.* **257,** E772-81 (1989).
3. Rhodes P *et al.* Adult-onset obesity reveals prenatal programming of glucose-insulin sensitivity in male sheep nutrient restricted during late gestation. *PLoS ONE* **4:** e7393 (2009).
4. Adam C.L., Findlay P.A., Aitken R.P., Milne J.S. & Wallace JM. In vivo changes in central and peripheral insulin sensitivity in a large animal model of obesity. *Endocrinology.* **153,** 3147-57 (2012)
5. Williams PJ *et al*. Hypertension and impaired renal function accompany juvenile obesity: the effect of prenatal diet. *Kidney International* **72**(3): 279 (1997).
6. Horn MA *et al*. Age-related divergent remodeling of the cardiac extracellular matrix in heart failure: collagen accumulation in the young and loss in the aged. *J Mol Cell Cardiol* **53**(1) 82 (2012).
7. Dodic M. *et al.* Altered cardiovascular haemodynamics and baroreceptor–heart rate reflex in adult sheep after prenatal exposure to dexamethasone. *Clinical Science* **97**(1) 103 (1999).
8. Dodic M. *et al.* Impaired cardiac functional reserve and left ventricular hypertrophy in adult sheep after prenatal dexamethasone exposure. *Circulation research* **89**(7) 623 (2001).
9. Gardner DS *et al.* Peri-implantation undernutrition programs blunted angiotensin II evoked baroreflex responses in young adult sheep. *Hypertension* **43**(6) 1290 (2004).
10. King A.J. *et al.* Hypertension caused by prenatal testosterone excess in female sheep. *Am J Physiol* **292** E1837 (2007).
11. Singh RR. Increased cardiovascular and renal risk is associated with low nephron endowment in aged females: an ovine model of fetal unilateral nephrectomy. *PLoS One* 7(8) e42400 (2012).
12. Sinclair, K.D. *et al*. DNA methylation, insulin resistance, and blood pressure in offspring determined by maternal periconceptional B vitamin and methionine status *PNAS.* **104**(49): 19351 (2007).
13. Kaler, J., Wassink, G.J. & Green, L.E. The inter- and intra-observer reliability of a locomotion scoring scale for sheep. *Vet. J.* **180,** 189–194 (2009).
14. Kellgren J.H. & Lawrence J.S. Radiological assessment of osteoarthrosis. *Ann. Rheumatic Dis*. **16,** 494e502 (1957).
15. Choi, I., Lee. J-H, Fisher, P. & Campbell KH. Caffeine treatment of ovine cytoplasts regulates gene expression and foetal development of embryos produced by somatic cell nuclear transfer. *Mol. Reprod. Dev*. **77,** 876-87 (2010).
